# Molecular epidemiology and evolutionary histories of human coronavirus OC43 and HKU1 among patients with upper respiratory tract infections in Kuala Lumpur, Malaysia

**DOI:** 10.1186/s12985-016-0488-4

**Published:** 2016-02-25

**Authors:** Maryam Nabiel Al-Khannaq, Kim Tien Ng, Xiang Yong Oong, Yong Kek Pang, Yutaka Takebe, Jack Bee Chook, Nik Sherina Hanafi, Adeeba Kamarulzaman, Kok Keng Tee

**Affiliations:** Department of Medicine, Faculty of Medicine, University of Malaya, Kuala Lumpur, Malaysia; AIDS Research Center, National Institute of Infectious Diseases, Toyama, Shinjuku-ku, Tokyo, Japan; School of Medicine, Yokohama City University, Yokohama, Kanagawa, Japan; Department of Primary Care Medicine, Faculty of Medicine, University of Malaya, Kuala Lumpur, Malaysia; Department of Medical Microbiology, Faculty of Medicine, University of Malaya, Kuala Lumpur, Malaysia

**Keywords:** Coronaviruses, Molecular epidemiology, Phylogenetics, Upper respiratory infection, Virus evolution

## Abstract

**Background:**

Despite the worldwide circulation of human coronavirus OC43 (HCoV-OC43) and HKU1 (HCoV-HKU1), data on their molecular epidemiology and evolutionary dynamics in the tropical Southeast Asia region is lacking.

**Methods:**

The study aimed to investigate the genetic diversity, temporal distribution, population history and clinical symptoms of betacoronavirus infections in Kuala Lumpur, Malaysia between 2012 and 2013. A total of 2,060 adults presented with acute respiratory symptoms were screened for the presence of betacoronaviruses using multiplex PCR. The spike glycoprotein, nucleocapsid and 1a genes were sequenced for phylogenetic reconstruction and Bayesian coalescent inference.

**Results:**

A total of 48/2060 (2.4 %) specimens were tested positive for HCoV-OC43 (1.3 %) and HCoV-HKU1 (1.1 %). Both HCoV-OC43 and HCoV-HKU1 were co-circulating throughout the year, with the lowest detection rates reported in the October-January period. Phylogenetic analysis of the spike gene showed that the majority of HCoV-OC43 isolates were grouped into two previously undefined genotypes, provisionally assigned as novel lineage 1 and novel lineage 2. Sign of natural recombination was observed in these potentially novel lineages. Location mapping showed that the novel lineage 1 is currently circulating in Malaysia, Thailand, Japan and China, while novel lineage 2 can be found in Malaysia and China. Molecular dating showed the origin of HCoV-OC43 around late 1950s, before it diverged into genotypes A (1960s), B (1990s), and other genotypes (2000s). Phylogenetic analysis revealed that 27.3 % of the HCoV-HKU1 strains belong to genotype A while 72.7 % belongs to genotype B. The tree root of HCoV-HKU1 was similar to that of HCoV-OC43, with the tMRCA of genotypes A and B estimated around the 1990s and 2000s, respectively. Correlation of HCoV-OC43 and HCoV-HKU1 with the severity of respiratory symptoms was not observed.

**Conclusions:**

The present study reported the molecular complexity and evolutionary dynamics of human betacoronaviruses among adults with acute respiratory symptoms in a tropical country. Two novel HCoV-OC43 genetic lineages were identified, warranting further investigation on their genotypic and phenotypic characteristics.

**Electronic supplementary material:**

The online version of this article (doi:10.1186/s12985-016-0488-4) contains supplementary material, which is available to authorized users.

## Background

Human coronaviruses are common cold viruses that are frequently found to be associated with acute upper respiratory tract infections (URTIs) [1]. According to the International Committee for Taxonomy of Viruses (ICTV), human coronavirus OC43 (HCoV-OC43) and HKU1 (HCoV-HKU1) belong to the betacoronavirus genus, a member of the *Coronaviridae* family. Coronaviruses contain the largest RNA genomes and have been established as one of the rapidly evolving viruses [2]. In addition to the high nucleotide substitution rates across the genome [3], the coronavirus genome is subjected to homologous recombination during viral replication, which is caused by RNA template switching mediated by the copy-choice mechanism [4, 5]. The genetic recombination of coronaviruses had possibly led to the emergence of lethal pathogens such as severe acute respiratory syndrome coronavirus (SARS-CoV) and Middle East respiratory syndrome coronavirus (MERS-CoV), which caused up to 50 % mortality in infected individuals [6–9]. Recombination events in the spike (S), nucleocapsid (N) and the RNA dependent RNA polymerase (RdRp) within the 1a gene of HCoV-OC43 and HCoV-HKU1 leading to the emergence of unique recombinant genotypes have been reported [10, 11].

Studies have shown that HCoV-OC43 is often associated with approximately 5 % of acute respiratory infections while the more recent HCoV-HKU1 is less prevalent [12, 13]. In humans, acute upper respiratory symptoms such as nasal congestion and rhinorrhea are relatively common in HCoV infections while sore throat and hoarseness of voice are less common, with cough usually associated with HCoV-OC43 infection [14]. In tropical countries, annual shift in the predominant genotype has been documented, with more cases of HCoV-OC43 and HCoV-HKU1 infections reported during the early months of the year [15]. Despite the clinical importance and socioeconomic impact of HCoV infections [16, 17], the prevalence, seasonality, clinical and phylogenetic characteristics of HCoVs remain largely unreported in the tropical region of Southeast Asia. Based on the S, N and 1a genes of HCoV-OC43 and HCoV-HKU1 isolated from Malaysia and also globally, we attempted to delineate the genetic history and the phylodynamic profiles of human betacoronaviruses HCoV-OC43 and HCoV-HKU1 using a suite of Bayesian phylogenetic tools. We also reported the emergence of two novel HCoV-OC43 lineages, in a cross-sectional study of patients presented with acute URTI in Malaysia.

## Methods

### Clinical specimens

A total of 2,060 consenting outpatient adults presented with symptoms of acute URTI were recruited at the Primary Care Clinics of University Malaya Medical Centre in Kuala Lumpur, Malaysia between March 2012 and February 2013. Prior to collection of nasopharyngeal swabs, demographic data such as age, gender and ethnicity were obtained. In addition, the severities of symptoms (sneezing, nasal discharge, nasal congestion, headache, sore throat, voice hoarseness, muscle ache and cough) were graded based on previously reported criteria [18–21]. The scoring scheme used had been validated earlier on the adult populations with common cold [19]. The nasopharyngeal swabs were transferred to the laboratory in universal transport media and stored in −80 °C.

### Molecular detection of HCoV-OC43 and HCoV-HKU1

Total nucleic acids were extracted from nasopharyngeal swabs using the magnetic beads-based protocols implemented in the NucliSENS easyMAG automated nucleic acid extraction system (BioMérieux, USA) [22, 23]. Specimens were screened for the presence of respiratory viruses using the xTAG Respiratory Virus Panel *FAST* multiplex RT-PCR assay (Luminex Molecular Diagnostics, USA) which can detect HCoV-OC43, HCoV-HKU1 and other respiratory viruses and subtypes [24].

### Genetic analysis of HCoV-OC43 and HCoV-HKU1

RNA from nasopharyngeal swabs positive for HCoV-OC43 and HCoV-HKU1 was reverse transcribed into cDNA using SuperScript III kit (Invitrogen, USA) with random hexamers (Applied Biosystems, USA). The partial S gene (S1 domain) [HCoV-OC43; 848 bp (24,030-24,865) and HCoV-HKU1; 897 bp (23,300-24,196)], complete N gene [HCoV-OC43; 1,482 bp (28,997-30, 478) and HCoV-HKU1; 1,458 bp (28,241-29,688)] and partial 1a (nsp3) gene [HCoV-OC43; 1,161 bp (6,168- 7,328) and HCoV-HKU1; 1,115 bp (6,472-7,586)] were amplified either by single or nested PCR, using 10 μM of the newly designed or previously described primers listed in Table 1. The PCR mixture (25 μl) contained cDNA, PCR buffer (10 mM Tris–HCl, 50 mM KCl, 3 mM MgCl, 0.01 % gelatin), 100 μM (each) deoxynucleoside triphosphates, Hi-Spec Additive and 4u/μl BIO-X-ACT Short DNA polymerase (BioLine, USA). The cycling conditions were as follows: initial denaturation at 95 °C for 5 min followed by 40 cycles of 94 °C for 1 min, 54.5 °C for 1 min, 72 °C for 1 min and a final extension at 72 °C for 10 min, performed in a C1000 Touch automated thermal cycler (Bio-Rad, USA). Nested/semi-nested PCR was conducted for each genetic region if necessary, under the same cycling conditions at 30 cycles. Purified PCR products were sequenced using the ABI PRISM 3730XL DNA Analyzer (Applied Biosystems, USA). The nucleotide sequences were codon-aligned with previously described complete and partial HCoV-OC43 and HCoV-HKU1 reference sequences retrieved from GenBank [11, 25–32].

**Table 1:** PCR primers of HCoV-OC43 and HCoV-HKU1

Target gene	HCoV	Primer	Location^a^	Sequence (5'-3')	Reference
Spike (S)	OC43	LPW 1261	24010-24029	Forward: CTRCTATARYTATAGGTAGT	[11]
		LPW 2094	24866-24887	Reverse: GCCCAAATTACCCAATTGTAGG	[11]
	HKU1	LPW 1832	23275-23299	Forward: TATGTTAATAAWACTTTGTATAGTG	[40]
		LPW 1866	24197-24218	Reverse: TACAATTGACAAGAACTAGAAG	[40]
Nucleocapsid (N)	OC43 & HKU1	βN-F	OC43: 28974-28996	Forward: GCTGTTTWTGTTAAGTCYAAAGT	this study
			HKU1: 28218-28240		
		βN-R	OC43: 30479-30501	Reverse: CATTCTGATAGAGAGTGCYTATY	this study
			HKU1: 29699-29721		
		βN-Fn	OC43: 29046-29069	Forward (nested): GCMTTGTTRAGARMTWAWATCTAA	this study
			HKU1: 28287-28310		
		βN-Rn	OC43: 30447-30466	Reverse (nested): GCGAGGGGTTACCACCWRRT	this study
			HKU1: 29671-29690		
1a	OC43	OC43-1aF	6145-6167	Forward: CTTTTGGTAAACCTGTTATATGG	this study
		OC43-1aR	7329-7351	Reverse: AGCTTAATAAAAGAGGCAATAAT	this study
		OC43-1aFn	6183-6199	Forward (semi-nested): GCTTCYCTCAATTCTTTAACAT	this study
	HKU1	HKU1-1aF	6448-6471	Forward: TTCTCTTACTTATTTTAATAAACC	this study
		HKU1-1aR	7587-7610	Reverse: CTTTATACATAGCAGTAACAACTA	this study

^a^Nucleotide location was determined based on the HCoV-OC43 ATCC VR-759 (AY585228) and HCoV-HKU1 (NC_06577) reference sequences

Maximum clade credibility (MCC) trees for the partial S (S1 domain), complete N and partial 1a (nsp3) genes were reconstructed in BEAST (version 1.7) [27, 33, 34]. MCC trees were generated using a relaxed molecular clock, assuming uncorrelated lognormal distribution under the general time-reversible nucleotide substitution model with a proportion of invariant sites (GTR + I) and a constant coalescent tree model. The Markov chain Monte Carlo (MCMC) run was set at 3 × 10^6^ steps long sampled every 10,000 state. The trees were annotated using Tree Annotator program included in the BEAST package, after a 10 % burn-in, and visualized in FigureTree (http://tree.bio.ed.ac.uk/software/Figuretree/). Neighbor joining (NJ) trees for the partial S (S1 domain), complete N and partial 1a (nsp3) genes were also reconstructed, using Kimura 2-parameter model in MEGA 5.1 [35]. The reliability of the branching order was evaluated by bootstrap analysis of 1000 replicates. In addition, to explore the genetic relatedness between HCoV-OC43 and HCoV-HKU1 genotypes, the pairwise genetic distances among sequences of the S gene were estimated. Inter- and intra-genotype nucleotide distances were estimated by the bootstrap analysis with 1000 replicates using MEGA 5.1. Such analysis has not been done for the N and the 1a genes because those regions were highly conserved across genotypes [10, 11, 32]. To test for the presence of recombination in HCoV-OC43, the S gene was subjected to pairwise distance-based bootscanning analysis using SimPlot version 3.5 [10, 36]. Established reference genomes for HCoV-OC43 genotype A (ATCC VR-759), B (87309 Belgium 2003), and C (HK04-01) were used as putative parental lineages, with a sliding window and step size of 160 bp and 20 bp, respectively. In addition, MaxChi recombination test [37] was performed in the Recombination Detection Program (RDP) version 4.0 [38]. In RDP the highest acceptable *p* value (the probability that sequences could share high identities in potentially recombinant regions by chance alone) was set at 0.05, with the standard multiple comparisons corrected using the sequential Bonferroni method with 1,000 permutations [39].

### Estimation of divergence time

The origin and divergence time (in calendar year) of HCoV-OC43 and HCoV-HKU1 genotypes were estimated using the MCMC approach as implemented in BEAST. Analyses were performed under the relaxed molecular clock with GTR + I nucleotide substitution models and constant-size and exponential demographic models. The MCMC analysis was computed at 3 × 10^6^ states sampled every 10,000 steps. The mean divergence time and the 95 % highest posterior density (HPD) regions were estimated, with the best-fitting models were selected by Bayes factor inference using marginal likelihood analysis implemented in Tracer (version 1.5) [33]. The evolutionary rate for S gene of betacoronaviruses (6.1 × 10^−4^ substitutions/site/year) reported previously was used for analysis [36].

### Statistical analysis

The association of HCoV-OC43 and HCoV-HKU1 infections with specific acute URTI symptoms and its severity (none, moderate and severe) as well as demographic data were evaluated using the Fisher’s exact test/Chi-square test carried out in the statistical package for the social sciences (SPSS, version 16; IBM Corp).

## Results

### Detection of HCoV-OC43 and HCoV-HKU1 in nasopharyngeal swabs

**Fig. 1:**
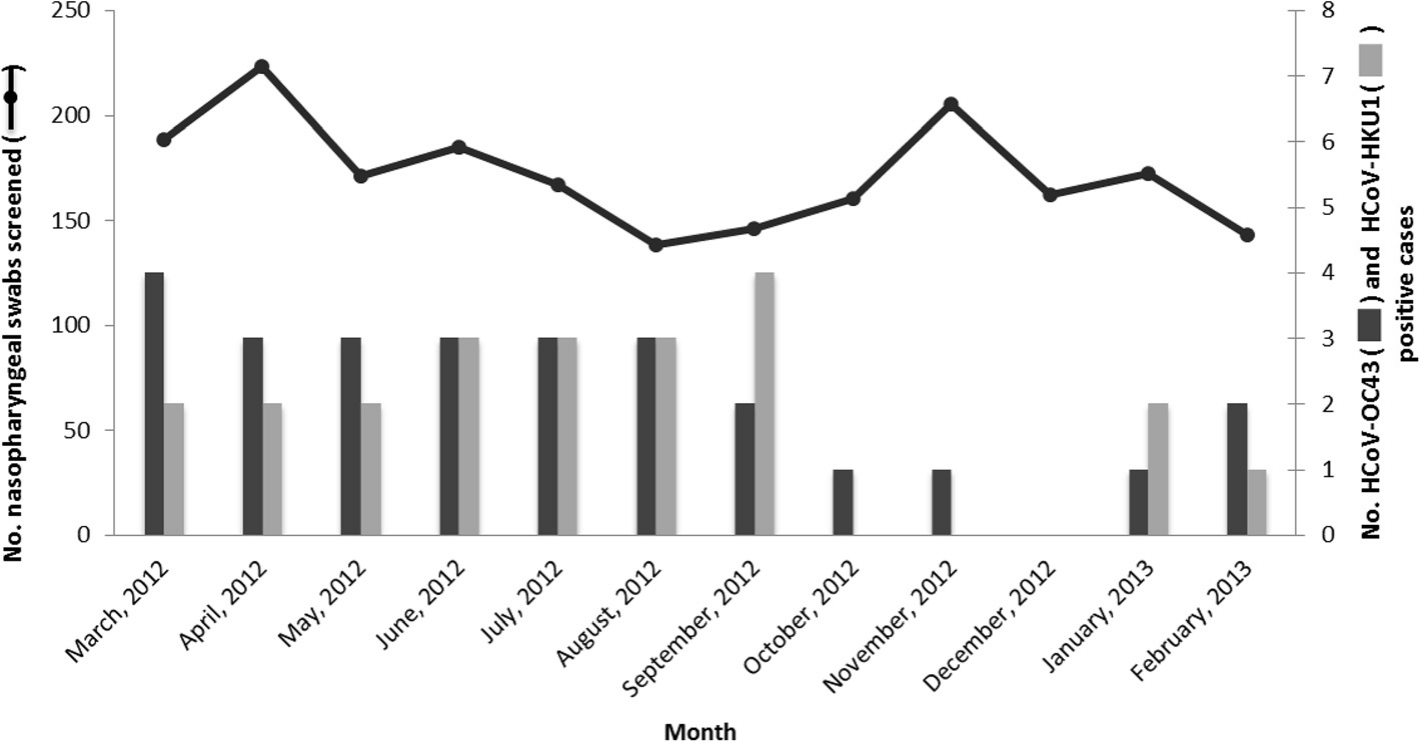
Annual distribution of HCoV-OC43 and HCoV-HKU1 among adults with acute in Malaysia. The monthly detection of HCoV-OC43 and HCoV-HKU1 (right axis, in bars) and the total number of nasopharyngeal swabs screened (left axis, in solid line) between March 2012 and February 2013 were shown

During the 12-month study period (March 2012 to February 2013), all nasopharyngeal swab specimens from 2,060 patients collected from Kuala Lumpur, Malaysia were screened for the presence of HCoV-OC43 and HCoV-HKU1 using multiplex RT-PCR method, in which a total of 48 (2.4 %) subjects were found positive for betacoronavirus. HCoV-OC43 and HCoV-HKU1 was detected in 26/2060 (1.3 %) and 22/2060 (1.1 %) patients, respectively, while no HCoV-OC43/HCoV-HKU1 co-infection was observed. Age, gender and ethnicity of the patients were summarized in Table 2. The median age of subjects infected with HCoV-OC43 and HCoV-HKU1 was 53.0 and 48.5, respectively. Both HCoV-OC43 and HCoV-HKU1 were co-circulating throughout the year, although lower numbers of HCoV-OC43 were detected between October 2012 and January 2013 while no HCoV-HKU1 was detected during these months (Fig. 1).

**Table 2:** Demographic data on 48 outpatients infected with human betacoronavirus in Kuala-Lumpur, Malaysia, 2012-2013

	HCoV-OC43 (*n* = 26)	HCoV-HKU1 (*n* = 22)	*P*-Value
Gender			
Male	11(42.3 %)	8(36.4 %)	0.77
Female	15(57.7 %)	14(63.6 %)	
Age			
<40	9(34.6 %)	10(45.4 %)	0.33
40–60	10(38.5 %)	4(18.2 %)	
>60	7(26.9 %)	8(36.4 %)	
Symptoms			
Sneezing	21(80.8 %)	14(63.6 %)	0.99
Nasal discharge	20(76.9 %)	19(86.4 %)	
Nasal congestion	19(73.1 %)	14(63.6 %)	
Headache	18(69.2 %)	16(72.7 %)	
Sore throat	16(61.5 %)	14(63.6 %)	
Hoarseness of voice	20(76.9 %)	18(81.8 %)	
Muscle ache	17(65.4 %)	14(63.6 %)	
Cough	23(88.5 %)	19(86.4 %)	
Ethnicity			
Malay	10(38.5 %)	10(45.4 %)	0.19
Chinese	3(11.5 %)	6(27.3 %)	
Indian	13(50.0 %)	6(27.3 %)	
Others	0(0.0 %)	0(0.0 %)

### Phylogenetic analysis of the S, N and 1a genes

**Fig. 2:**
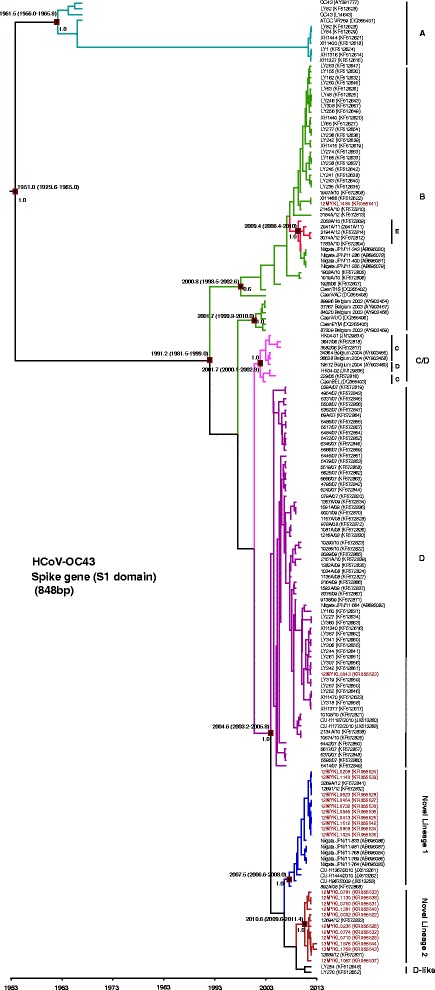
Maximum clade credibility (MCC) tree of HCoV-OC43 genotypes. Estimation of the time of the most recent common ancestors (tMRCA) with 95 % highest posterior density (95 % HPD) of HCoV-OC43 genotypes based on the spike gene (S1 domain) (848 bp). Data were analyzed under relaxed molecular clock with GTR + I substitution model and a constant size coalescent model implemented in BEAST. The Malaysian HCoV-OC43 isolates obtained in this study were color-coded and the HCoV-OC43 genotypes (**a**) to (**e**) as well as novel lineages 1 and 2 were indicated. The MCC posterior probability values were indicated on the nodes of each genotype

The partial S (S1 domain), complete N and partial 1a (nsp3) genes of 23 HCoV-OC43 isolates were successfully sequenced, while another three xTAG-positive HCoV-OC43 isolates could not be amplified, probably due to low viral copy number in these specimens. Based on the phylogenetic analysis of the S gene, one subject (1/23, 4.3 %) was grouped with HCoV-OC43 genotype B reference sequences while another subject (1/23, 4.3 %) was grouped with HCoV-OC43 genotype D sequences. The remaining 21 isolates formed two phylogenetically discrete clades that were distinct from other previously established genotypes A, B, C, D (genotype D is a recombinant lineage that is not readily distinguished from genotype C in the S and N phylogenetic trees) and E [11, 32] (Fig. 2 and Additional file 1: Figure S1). Of the 21 isolates, ten isolates have formed a cluster with other recently reported isolates from Japan, Thailand and China [31, 32] supported by the posterior probability value of 1.0 and bootstrap value of 36 % at the internal tree node of the MCC and NJ trees, respectively with intra-group pairwise genetic distance of 0.003 ± 0.001. These isolates were provisionally designated as novel lineage 1. Spatial structure was observed within novel lineage 1, with an isolate from China sampled in year 2008 located at the base of the phylogeny. Moreover, another eleven HCoV-OC43 isolates have formed a second distinct cluster supported by significant posterior probability and bootstrap values at the internal tree node (1.0 and 98 %, respectively) and intra-group pairwise genetic distance of 0.004 ± 0.001. The cluster contained Malaysian and Chinese isolates [32] only, and was denoted as novel lineage 2. Based on the phylogenetic inference of the conserved N gene, only one subject was grouped with the genotype B reference in concordance with the S gene (Additional file 2: Figure S2). Unlike the phylogenetic inference of the S gene, the remaining 22 isolates were seen intermingled with each other forming a single cluster together with isolates indicated as novel lineages 1 and 2 in the S gene, in addition to one genotype D strain. It is however important to note that the tree resolution was poor, due primarily to the lack of the N gene reference sequences in the public database. On the other hand, phylogenetic analysis of the 1a (nsp3) gene (Additional file 3: Figure S3) revealed that all except genotype A could not be differentiated clearly within this region, due mainly to the low genetic diversity between genotypes. The limited number of 1a reference sequences available in the public database could have also resulted in a poor 1a tree topology. In addition, phylogenetic trees of previously described complete and partial S gene sequences as well as partial 1a (nsp3) and complete RdRp gene sequences were reconstructed to further confirm the reliability of the partial S1 and nsp3 for identification of HCoV-OC43 genotypes (Additional file 4: Figure S4 and Additional file 5: Figure S5).

To assess the diversity between HCoV-OC43 genotypes, inter-genotype pairwise genetic distance was estimated for the S gene, listed in Table 3. Using the oldest genotype as reference i.e. genotype A, genetic variation between genotype A and genotypes B to E was 2.2–2.7 %. Genetic distance between novel lineages 1 and 2 compared to genotype A was 3.2 % and 3.1 %, respectively, higher than that of other established genotypes. Taken together, the distinct inter-genotype genetic variations of the two novel lineages 1 and 2 against other previously established genotypes corroborated with the MCC inference (Fig. 2) in which both lineages formed distinct phylogenetic topologies.

**Table 3:** Genetic distance among HCoV-OC43 and HCoV-HKU1 genotypes in the spike gene

	HCoV	Genetic distance						
OC43		genotype A	genotype B	genotype C	genotype D	genotype E	Novel lineage 1	Novel lineage 2
	genotype A	-						
	genotype B	2.7	-					
	genotype C	2.2	1.5	-				
	genotype D	2.7	1.8	0.8	-			
	genotype E	2.5	0.9	1.2	1.6	-		
	Novel lineage 1	3.2	2.0	1.3	0.7	1.9	-	
	Novel lineage 2	3.1	2.9	1.8	1.4	2.6	1.7	-
HKU1		genotype A	genotype B	genotype C				
	genotype A	-						
	genotype B	15.7	-					
	genotype C	15.2	1.3	-				

Pairwise genetic distances are expressed in percentage (%) difference

**Fig. 3:**
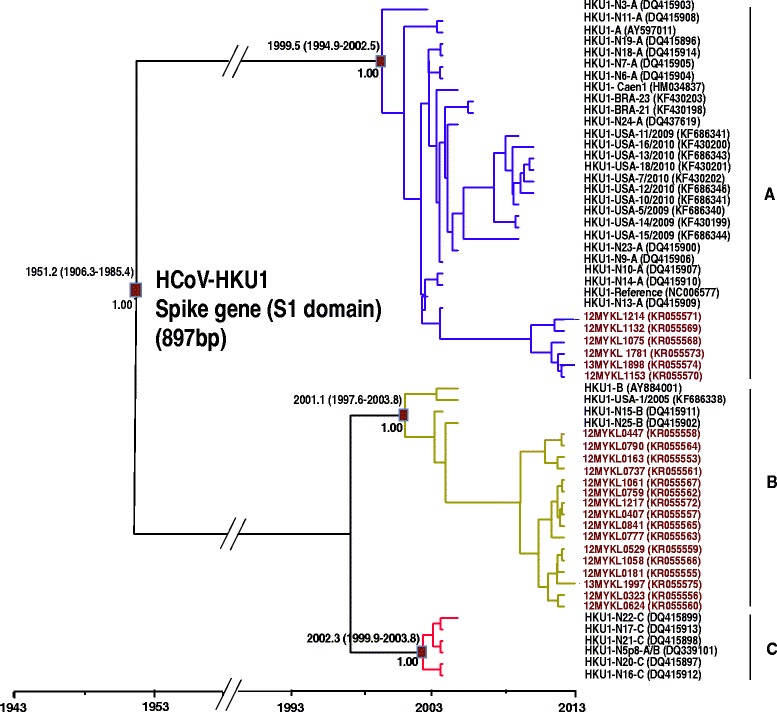
Maximum clade credibility (MCC) tree of HCoV-HKU1 genotypes. Estimation of the time of the most recent common ancestors (tMRCA) with 95 % highest posterior density (95 % HPD) of HCoV-HKU1 genotypes based on the spike gene (S1 domain) (897 bp). Data were analyzed under relaxed molecular clock with GTR + I substitution model and a constant size coalescent model implemented in BEAST. The Malaysian HCoV-HKU1isolates obtained in this study were color-coded and the HCoV-HKU1 genotypes (**a**) to (**c**) were indicated. The MCC posterior probability values were indicated on the nodes of each genotype

On the other hand, phylogenetic analysis of 22 HCoV-HKU1 S and N genes indicated the predominance of HCoV-HKU1 genotype B (72.7 %, 16/22), followed by HCoV-HKU1 genotype A (27.3 %, 6/22) (Fig. 3, Additional file 6: Figure S6 and Additional file 7: Figure S7). Interestingly, the S and N genes of HCoV-HKU1 were equally informative for genotype assignment, while genotypes A, B and C were less distinctive based on the 1a gene phylogenetic analysis due to the high genetic conservation within this region (Additional file 8: Figure S8). Inter-genotype genetic diversity among HCoV-HKU1 genotypes showed that genotype A was more genetically diverse than genotypes B and C based on the genetic data of the S gene (Table 3). The difference in genetic distance between genotype A and genotypes B and C was 15.2–15.7 %, while the difference in genetic distance between genotypes B and C was 1.3 %.

**Fig. 4:**
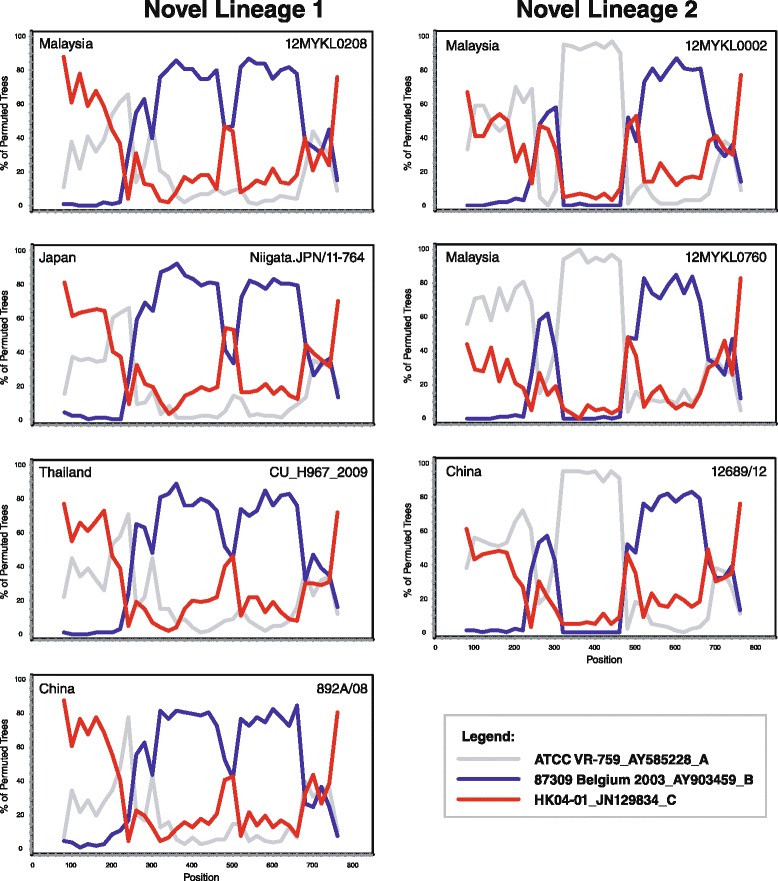
Recombination analyses of HCoV-OC43 novel lineages 1 and 2. Reference strains of HCoV-OC43 genotype A (ATCC VR-759), B (87309 Belgium 2003), and C (HK04-01) were used as the putative parental strains. The bootstrap values were plotted for a window of 160 bp moving in increments of 20 bp along the alignment. Samples 12MYKL0208, Niigata.JPN/11-764, CU-H967_2009, 892A/08 were used as representative sequences for novel lineage 1 in addition to 12MYKL0002, 12MYKL0760 and 12689/12 isolates as representatives for novel lineage 2

Evidence of possible recombination was observed in the S gene of novel lineage 1, involving genotypes B and C (Fig. 4). All isolates within novel lineage 1 showed similar recombination structures (representative isolates from Malaysia (12MYKL0208), Japan (Niigata.JPN/11-764), Thailand (CU-H967_2009) and China (892A/08) were shown). Similarly, sign of possible recombination was noticed within novel lineage 2 (Fig. 4). All Malaysian and Chinese isolates showed similar recombination structures in the S gene involving genotypes A and B (12MYKL0002, 12MYKL0760 and 12689/12 representative sequences were shown). Moreover, using the aforementioned putative parental and representative strains, MaxChi analysis of the novel lineages 1 and 2 isolates supported the hypothesis of recombination in the S gene (*p* < 0.05) (Additional file 9: Figure S9). Taken together, the emergence of novel lineage 1 and novel lineage 2 in these Asian countries was likely to be driven by natural recombination events.

### Estimation of divergence times

The divergence times of HCoV-OC43 and HCoV-HKU1 were estimated using the coalescent-based Bayesian relaxed molecular clock under the constant and exponential tree models (Fig. 2 and Fig. 3; Table 4). The newly estimated mean evolutionary rate for the S gene of HCoV-OC43 was 7.2 (5.0 – 9.3) × 10^−4^ substitutions/site/year. On the other hand, the evolutionary rate for the S gene of HCoV-HKU1 was newly estimated at 6.2 (4.2–7.8) × 10^−4^ substitutions/site/year. These estimates were comparable to previous findings of 6.1–6.7 × 10^−4^ substitutions/site/year for the S gene reported elsewhere [11].

**Table 4:** Evolutionary characteristics of HCoV-OC43 and HCoV-HKU1 genotypes

Subtype-gene evolutionary rate^a^	Genotype	tMRCA^b^
OC43-Spike 7.2 (5.2–9.4)		
	all genotypes	1952.2 (1931.0–1965.2)
	genotype A	1961.8 (1955.1–1966.0)
	genotype B	1991.0 (1981.4–1999.0)
	genotype C/D	2001.7 (2000.1–2002.9)
	genotype D	2004.5 (2003.3–2005.8)
	genotype E	2009.3 (2008.3–2010.0)
	novel lineage 1	2007.5 (2006.6–2008.0)
	novel lineage 2	2010.5 (2009.5–2011.4)
HKU1-Spike 6.2 (4.5–8.0)		
	all genotypes	1957.2 (1920.3–1987.5)
	genotype A	1999.4 (1994.8–2002.5)
	genotype B	2001.2 (1997.6–2003.6)
	genotype C	2002.3 (1999.8–2003.8)
HKU1-Nucleocapsid 4.3 (2.8–5.8)		
	all genotypes	1962.0 (1915.1–1994.8)
	genotype A	1986.8 (1970.8–1999.0)
	genotype B	2002.2 (1999.4–2002.2)
	genotype C	2002.3 (2000.1–2003.8)

^a^Estimated mean rates of evolution expressed as 10^−4^ nucleotide substitutions/site/year under a relaxed molecular clock with GTR + I substitution model and an Exponential tree model. The 95 % highest posterior density (HPD) confidence intervals are included in parentheses

^b^Mean time of the most common ancestor (tMRCA, in calendar year). The 95 % highest posterior density (HPD) confidence intervals are indicated

Based on these evolutionary estimates of the S gene, the common ancestor of HCoV-OC43 was dated back to the 1950s. Divergence time of genotype A was dated back to early 1960s, followed by genotype B around 1990s. Interestingly, genotypes C, D, E, and novel lineages 1 and 2 were all traced back to the 2000s (Fig. 2). Moreover, the common ancestor of HCoV-HKU1 was traced back to early 1950s, as estimated from the S gene. Subsequently, HCoV-HKU1 continued to diverge further into distinctive genotypes (A-C). Genotype A was dated to the late 1990 and genotypes B and C were both traced back to early 2000s (Fig. 3). Bayes factor analysis showed insignificant differences (Bayes factor <3.0) between the constant and exponential coalescent models of demographic analysis. Divergence times generated using the exponential tree model were slightly (but not significantly) different from those estimated using the constant coalescent model (Table 4). Of note, HCoV-OC43 and HCoV-HKU1 genotype assignments were less distinctive within the N and 1a genes (as compared to the S gene); these regions were therefore deemed unsuitable for divergence time estimations in this study.

### Clinical symptoms assessment

The type of URTI symptoms (sneezing, nasal discharge, nasal congestion, headache, sore throat, hoarseness of voice, muscle ache and cough) and their severities during HCoV-OC43 and HCoV-HKU1 infections were analyzed. Fisher’s exact test analysis suggested that the severity of symptoms was not significantly associated with HCoV-OC43 and HCoV-HKU1 infections (*p* values > 0.05), this is due to the fact that the majority (61 % and 55 %) of the patients infected with HCoV-OC43 and HCoV-HKU1 respectively were presented with at least one respiratory symptom at moderate level of symptom severity. In addition, no significant association between HCoV-OC43 and HCoV-HKU1 genotypes with disease severity was observed.

## Discussion

In the present cohort, over 2000 patients with URTI symptoms were recruited and screened, of whom 1.3 % (26/2060) and 1.1 % (22/2060) of the subjects were infected with HCoV-OC43 and HCoV-HKU1, respectively. These estimates corroborate with the previously reported average incidence of HCoV-OC43 and HCoV-HKU1 at 0.2–4.3 % and 0.3–4.4 %, respectively [12, 15, 40–45]. Although HCoV-OC43 and HCoV-HKU1 are not as common as other respiratory viruses, several studies have reported an elevated incidence of HCoV-OC43 (up to 67 %) due to sporadic outbreaks with fatality rate up to 8 % [46, 47]. This 12-month study showed that HCoV-OC43 and HCoV-HKU1 infections were frequently detected during March 2012 to September 2012 and decreased thereafter, in line with findings reported from other tropical Southeast Asian country [15]. However, such patterns differ from that in temperate areas where the prevalence peaks during winter seasons, but few or no detections in the summer [43]. It is also important to note that the study was performed in a relatively short duration, therefore limiting the epidemiological and disease trend comparison with reports from other countries.

Phylogenetic inference based on the S gene of HCoV-OC43 suggested the emergence of two potentially novel genotypes (designated as novel lineage 1 and novel lineage 2), supported by phylogenetic evidence and shared recombination structures. The relatively low mean intra-cluster genetic variation reflects the high intra-genotype genetic homogeneity of each novel lineage. Inter-genotype genetic distances between HCoV-OC43 genotypes further supported that the novel lineages 1 and 2 are distinct from the previously described genotypes [11, 17, 32] in which the genetic distances between each of these two genotypes and the others were notably high (up to 3.2 %) (Table 3). Phylogenetic analysis also revealed that novel lineage 1 includes isolates from Malaysia, Thailand, China and Japan while novel lineage 2 isolates are all from Malaysia and China. Spatiotemporal characteristic observed within the novel lineage 1 phylogeny (Fig. 2) may suggest the origin of this lineage in China, before it spread to other regions in the East and Southeast Asia. In order to clearly define the genetic characteristic of the putative novel lineages 1 and 2 (and also any other isolates with discordant phylogenetic patterns), complete genome sequencing and phylogenetic analysis need to be carried out.

Based on the newly estimated substitution rates, the divergence times for HCoV-OC43 and HCoV-HKU1 were phylogenetically inferred. Interestingly, although HCoV-OC43 was the first human coronavirus discovered in 1965 [48, 49], and the HCoV-HKU1 was first described much later in 2005 [50], the S gene analysis of HCoV-OC43 and HCoV-HKU1 revealed that the respective common ancestors of both viruses have emerged since 1950s. Furthermore, the divergence times of HCoV-OC43 genotypes predicted in this study are comparable to those described in previous studies [11, 27]. Phylogenetic, recombination and molecular clock analysis suggest the emergence of novel lineages 1 and 2 around the mid-2000s and late 2000s, respectively, probably by natural recombination events involving genotypes B and C (for lineage 1) and genotypes A and B (for lineage 2).

Human coronaviruses are progressively recognized as respiratory pathogens associated with an increasing range of clinical outcomes. Our results indicated that most patients infected with HCoV-OC43 and HCoV-HKU1 were presented with moderate respiratory symptoms (data not shown) in accordance with previously reported clinical results [16, 51–53] where they were recognized as common cold viruses associated with URTI symptoms.

## Conclusions

In conclusion, epidemiological and evolutionary dynamics investigation revealed the genetic complexity of human betacoronaviruses HCoV-OC43 and HCoV-HKU1 infections in Malaysia, identifying two potentially novel HCoV-OC43 lineages among adults with acute respiratory tract infections. The reported findings warrant continuous molecular surveillance in the region, and detailed genotypic and phenotypic characterization of the novel betacoronavirus lineages.

## Declarations

### Ethics statement

The study was approved by the University of Malaya Medical Ethics Committee (MEC890.1). Standard, multilingual consent forms allowed by the Medical Ethics Committee were used. Written consents were obtained from all study participants.

### Consent for publication

Not applicable.

### Availability of data and materials

HCoV-OC43 and HCoV-HKU1 nucleotide sequences generated in the study are available in GenBank under the accession numbers KR055512-KR055644.

## Additional files

Additional file 1: Figure S1. Phylogenetic analysis of the HCoV-OC43 spike gene (S1 domain). Trees were reconstructed using neighbor-joining method. Bootstrap values were calculated from 1,000 trees. The scale bar of individual tree was indicated in substitutions per site, using Kimura 2-parameter model in MEGA (version 5.1) to estimate pair-wise evolutionary distance. The Malaysian isolates obtained in this study were color-coded and the HCoV-OC43 genotypes A to E as well as novel lineages 1 and 2 were indicated. (PDF 242 kb) Additional file 2: Figure S2. Phylogenetic analysis of the HCoV-OC43 nucleocapsid gene. Trees were reconstructed using neighbor-joining method. Bootstrap values were calculated from 1,000 trees. Bootstrap values of greater than 70 % were indicated on the branch nodes. The scale bar of individual tree was indicated in substitutions per site, using Kimura 2-parameter model in MEGA (version 5.1) to estimate pair-wise evolutionary distance. The Malaysian isolates obtained in this study were color-coded and the HCoV-OC43 genotypes A to E as well as novel lineages 1 and 2 were indicated. Each HCoV-OC43 sequence was assigned to its proper genotype based on the S1 phylogenetic analysis. NL1= novel lineage 1. (PDF 253 kb) Additional file 3: Figure S3. Phylogenetic analysis of the HCoV-OC43 1a gene (nsp3). Tree was reconstructed using neighbor-joining method. Bootstrap values were calculated from 1,000 trees. Bootstrap values of greater than 70% were indicated on the branch nodes. The scale bar of individual tree was indicated in substitutions per site, using Kimura 2-parameter model in MEGA (version 5.1) to estimate pair-wise evolutionary distance. The Malaysian isolates obtained in this study were color-coded. Each HCoV-OC43 sequence was assigned to its proper genotype based on the S1 phylogenetic analysis. (PDF 284 kb) Additional file 4: Figure S4. Phylogenetic analysis of the HCoV-OC43 complete and partial S gene. Trees were reconstructed using neighbor-joining method. Bootstrap values were calculated from 1,000 trees. Bootstrap values of greater than 70 % were indicated on the branch nodes. The scale bar of individual tree was indicated in substitutions per site, using Kimura 2-parameter model in MEGA (version 5.1) to estimate pair-wise evolutionary distance. NL1= novel lineage 1, NL2= novel lineage 2. (PDF 208 kb) Additional file 5: Figure S5. Phylogenetic analysis of the HCoV-OC43 1a (nsp3) and RdRp gene. Trees were reconstructed using neighbor-joining method. Bootstrap values were calculated from 1,000 trees. Bootstrap values of greater than 70 % were indicated on the branch nodes. The scale bar of individual tree was indicated in substitutions per site, using Kimura 2-parameter model in MEGA (version 5.1) to estimate pair-wise evolutionary distance. (PDF 119 kb) Additional file 6: Figure S6. Phylogenetic analysis of the HCoV-HKU1 spike gene (S1 domain). Tree was reconstructed using neighbor-joining method. Bootstrap values were calculated from 1,000 trees. Bootstrap values of greater than 70% were indicated on the branch nodes. The scale bar of individual tree was indicated in substitutions per site, using Kimura 2-parameter model in MEGA (version 5.1) to estimate pair-wise evolutionary distance. The Malaysian isolates obtained in this study were color-coded. (PDF 360 kb) Additional file 7: Figure S7. Phylogenetic analysis of the HCoV-HKU1 nucleocapsid gene. Tree was reconstructed using neighbor-joining method. Bootstrap values were calculated from 1,000 trees. Bootstrap values of greater than 70 % were indicated on the branch nodes. The scale bar of individual tree was indicated in substitutions per site, using Kimura 2-parameter model in MEGA (version 5.1) to estimate pair-wise evolutionary distance. The Malaysian isolates obtained in this study were color-coded. (PDF 347 kb) Additional file 8: Figure S8. Phylogenetic analysis of the HCoV-HKU1 1a gene (nsp3). Trees was reconstructed using neighbor-joining method. Bootstrap values were calculated from 1,000 trees. Bootstrap values of greater than 70% were indicated on the branch nodes. The scale bar of individual tree was indicated in substitutions per site, using Kimura 2-parameter model in MEGA (version 5.1) to estimate pair-wise evolutionary distance. The Malaysian isolates obtained in this study were color-coded. (PDF 133 kb) Additional file 9: Figure S9. Recombination analysis in HCoV-OC43 novel lineages 1 and 2. Analysis of the partial S gene was carried out using the MaxChi method in RDP. The x-axis gives the nucleotide positions of the alignment, whereas the y-axis presents the particular test statistics. Peaks in the log *P* of χ^2^ values in the MaxChi test marks potential points of recombination. Dashed lines represent p value cut-offs: uncorrected (lower line) and corrected for multiple comparisons (upper line) at the 0.05 level. (PDF 330 kb)

## Abbreviations

GTR + I: general time-reversible nucleotide substitution model with invariant sites
HCoV-HKU1: human coronavirus HKU1
HCoV-OC43: human coronavirus OC43
HPD: highest posterior density
ICTV: International Committee for Taxonomy of Viruses
MCC: maximum clade credibility
MCMC: Markov chain Monte Carlo
MERS-CoV: Middle East respiratory syndrome coronavirus
NJ: neighbor joining
RdRp: RNA dependent RNA polymerase
SARS-CoV: severe acute respiratory syndrome coronavirus
tMRCA: time of the most recent common ancestors
URTI: upper respiratory tract infection
