# Supplementary figures and images for: Molecular epidemiology and evolutionary histories of human coronavirus OC43 and HKU1 among patients with upper respiratory tract infections in Kuala Lumpur, Malaysia
Source: Virol J. 2016 Feb 25;13:33. doi: 10.1186/s12985-016-0488-4 (PMC4766700; doi:10.1186/s12985-016-0488-4)

HCoV-OC43

Spike gene (S1 domain)  
(848bp)

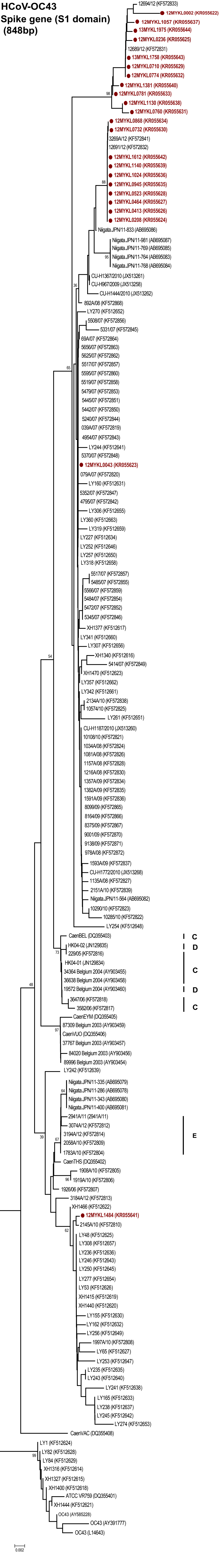

Novel Lineage 2

Novel Lineage 1

D-like

D

D-like

C/D

E

B

A

0.002

Supplement: Additional file 1: Figure S1. — Phylogenetic analysis of the HCoV-OC43 spike gene (S1 domain). Trees were reconstructed using neighbor-joining method. Bootstrap values were calculated from 1,000 trees. The scale bar of individual tree was indicated in substitutions per site, using Kimura 2-parameter model in MEGA (version 5.1) to estimate pair-wise evolutionary distance. The Malaysian isolates obtained in this study were color-coded and the HCoV-OC43 genotypes A to E as well as novel lineages 1 and 2 were indicated. (PDF 242 kb) [file 12985_2016_488_MOESM1_ESM.pdf]

**HCoV-OC43**  
**Nucleocapsid gene**  
**(1484bp)**

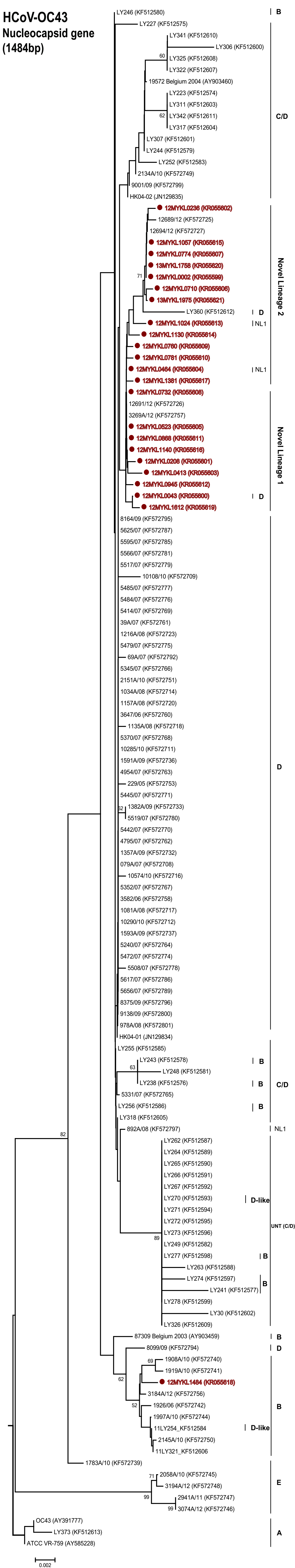

Supplement: Additional file 2: Figure S2. — Phylogenetic analysis of the HCoV-OC43 nucleocapsid gene. Trees were reconstructed using neighbor-joining method. Bootstrap values were calculated from 1,000 trees. Bootstrap values of greater than 70 % were indicated on the branch nodes. The scale bar of individual tree was indicated in substitutions per site, using Kimura 2-parameter model in MEGA (version 5.1) to estimate pair-wise evolutionary distance. The Malaysian isolates obtained in this study were color-coded and the HCoV-OC43 genotypes A to E as well as novel lineages 1 and 2 were indicated. Each HCoV-OC43 sequence was assigned to its proper genotype based on the S1 phylogenetic analysis. NL1= novel lineage 1. (PDF 253 kb) [file 12985_2016_488_MOESM2_ESM.pdf]

HCoV-OC43  
1a (nsp3) gene (1161bp)

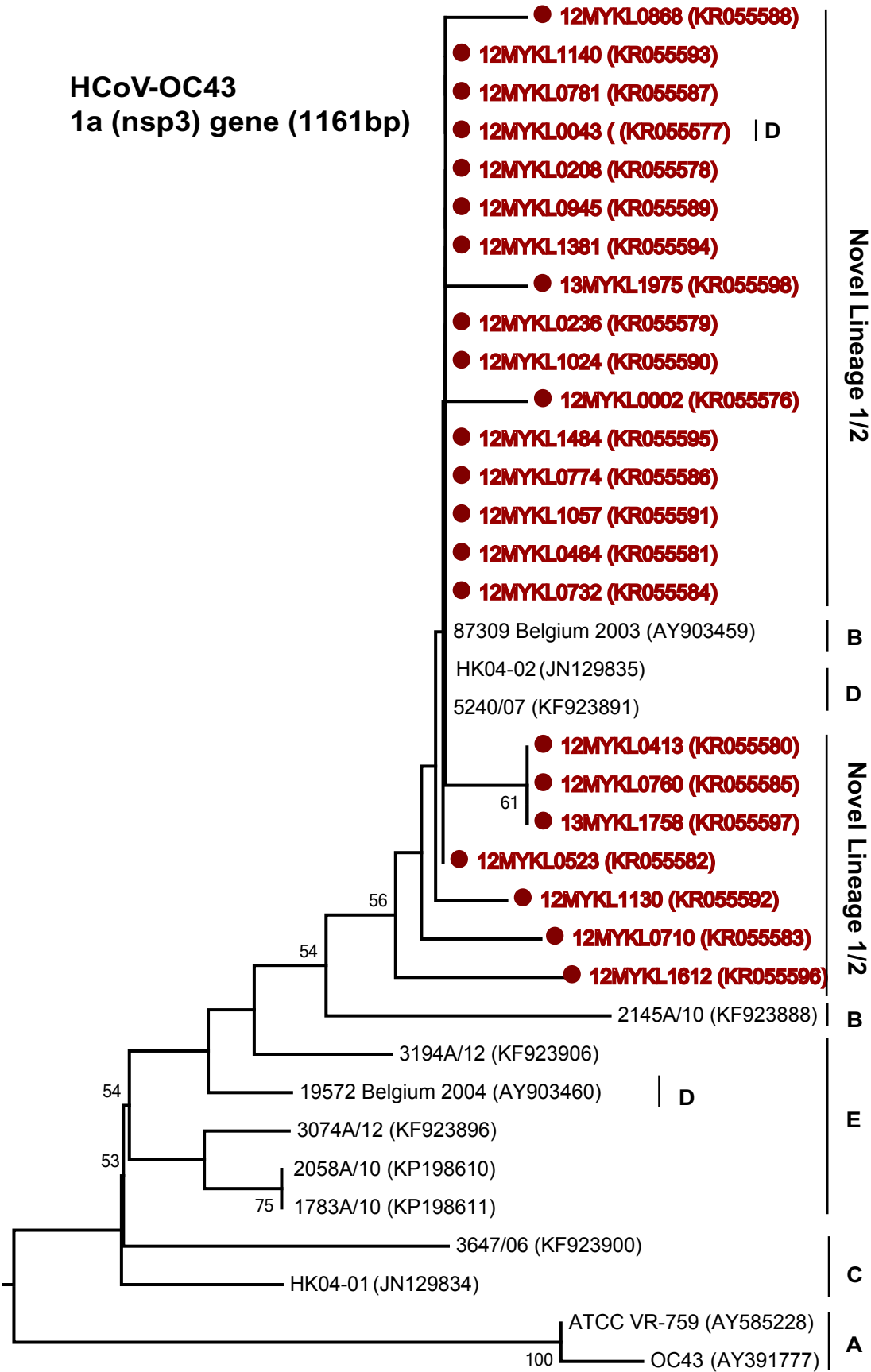

0.001

Supplement: Additional file 3: Figure S3. — Phylogenetic analysis of the HCoV-OC43 1a gene (nsp3). Tree was reconstructed using neighbor-joining method. Bootstrap values were calculated from 1,000 trees. Bootstrap values of greater than 70% were indicated on the branch nodes. The scale bar of individual tree was indicated in substitutions per site, using Kimura 2-parameter model in MEGA (version 5.1) to estimate pair-wise evolutionary distance. The Malaysian isolates obtained in this study were color-coded. Each HCoV-OC43 sequence was assigned to its proper genotype based on the S1 phylogenetic analysis. (PDF 284 kb) [file 12985_2016_488_MOESM3_ESM.pdf]

HCoV-OC43

Spike gene (4059bp)

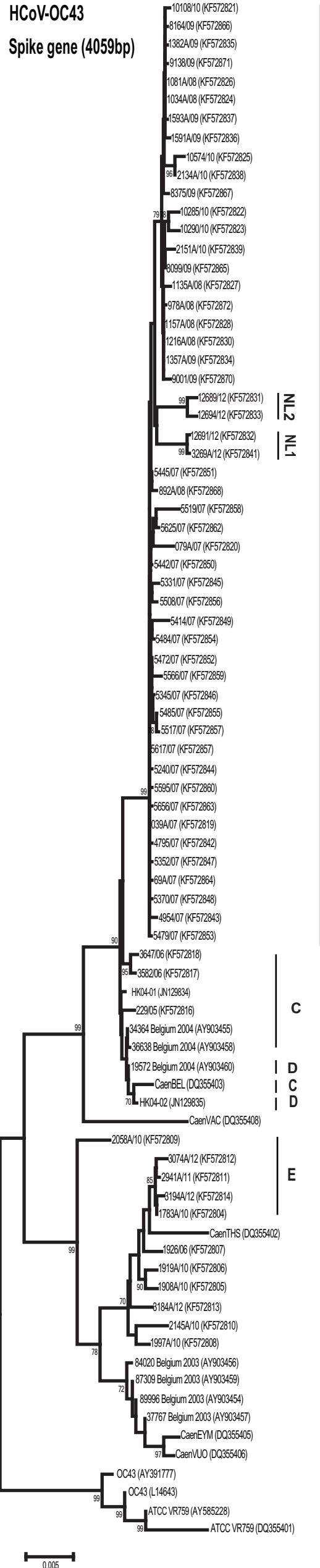

HCoV-OC43

Spike gene (S1 domain) (848bp)

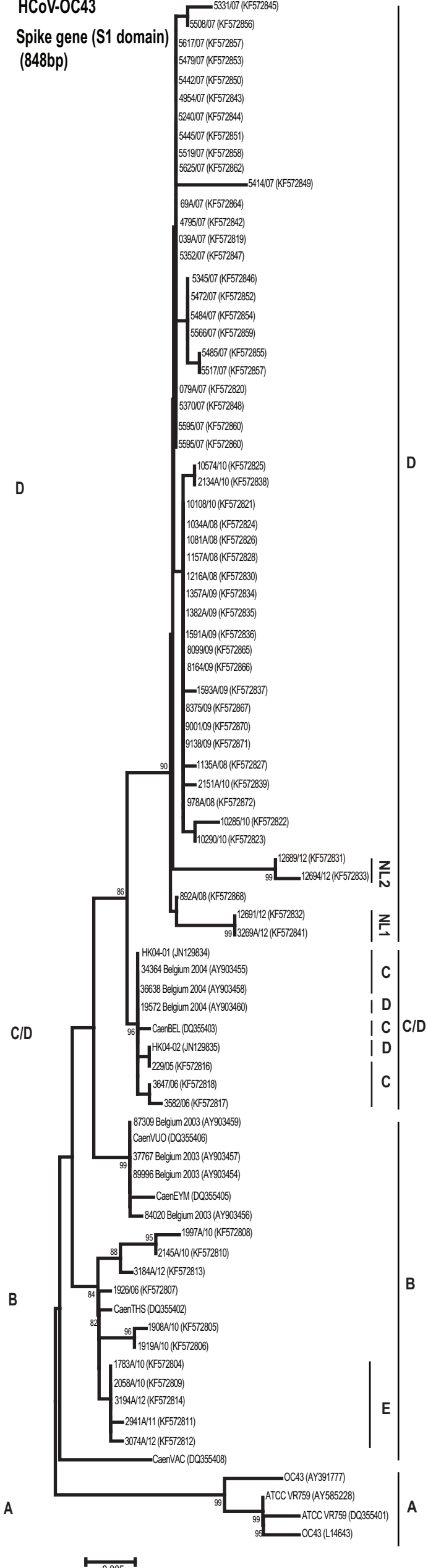

Supplement: Additional file 4: Figure S4. — Phylogenetic analysis of the HCoV-OC43 complete and partial S gene. Trees were reconstructed using neighbor-joining method. Bootstrap values were calculated from 1,000 trees. Bootstrap values of greater than 70 % were indicated on the branch nodes. The scale bar of individual tree was indicated in substitutions per site, using Kimura 2-parameter model in MEGA (version 5.1) to estimate pair-wise evolutionary distance. NL1= novel lineage 1, NL2= novel lineage 2. (PDF 208 kb) [file 12985_2016_488_MOESM4_ESM.pdf]

**HCoV-OC43**  
**1a (nsp3) gene (1161bp)**

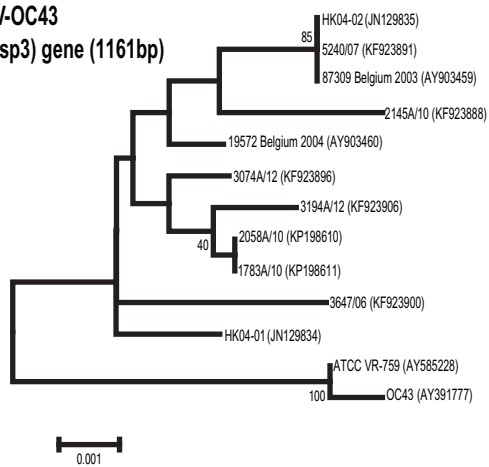

**HCoV-OC43**  
**RdRp gene (2783 bp)**

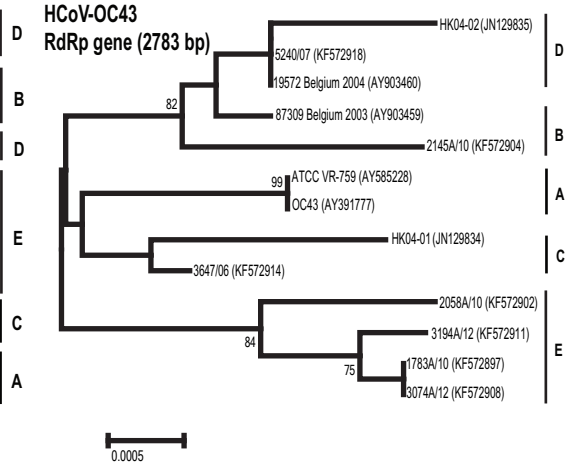

Supplement: Additional file 5: Figure S5. — Phylogenetic analysis of the HCoV-OC43 1a (nsp3) and RdRp gene. Trees were reconstructed using neighbor-joining method. Bootstrap values were calculated from 1,000 trees. Bootstrap values of greater than 70 % were indicated on the branch nodes. The scale bar of individual tree was indicated in substitutions per site, using Kimura 2-parameter model in MEGA (version 5.1) to estimate pair-wise evolutionary distance. (PDF 119 kb) [file 12985_2016_488_MOESM5_ESM.pdf]

HCoV-HKU1  
Spike gene (S1 domain) (897bp)

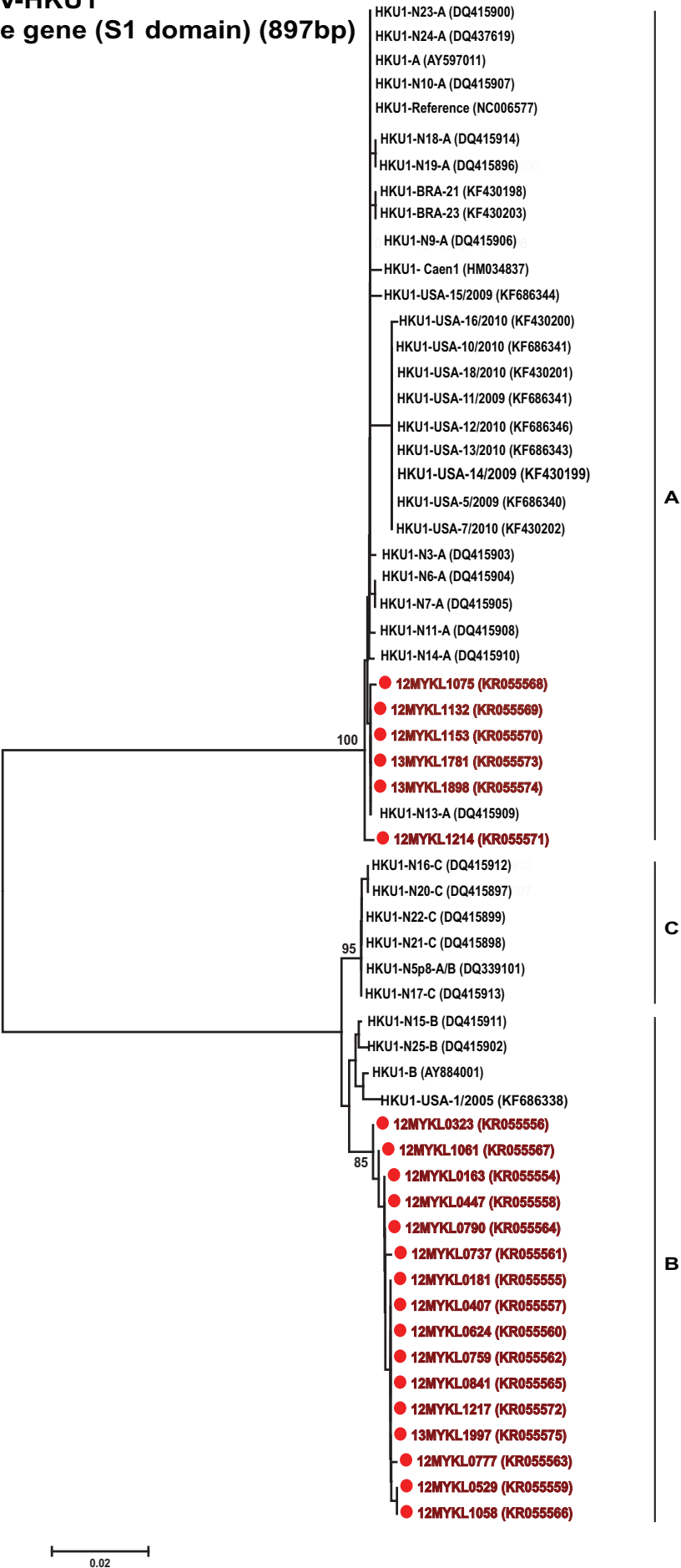

Supplement: Additional file 6: Figure S6. — Phylogenetic analysis of the HCoV-HKU1 spike gene (S1 domain). Tree was reconstructed using neighbor-joining method. Bootstrap values were calculated from 1,000 trees. Bootstrap values of greater than 70% were indicated on the branch nodes. The scale bar of individual tree was indicated in substitutions per site, using Kimura 2-parameter model in MEGA (version 5.1) to estimate pair-wise evolutionary distance. The Malaysian isolates obtained in this study were color-coded. (PDF 360 kb) [file 12985_2016_488_MOESM6_ESM.pdf]

HCoV-HKU1  
Nucleocapsid gene (1458bp)

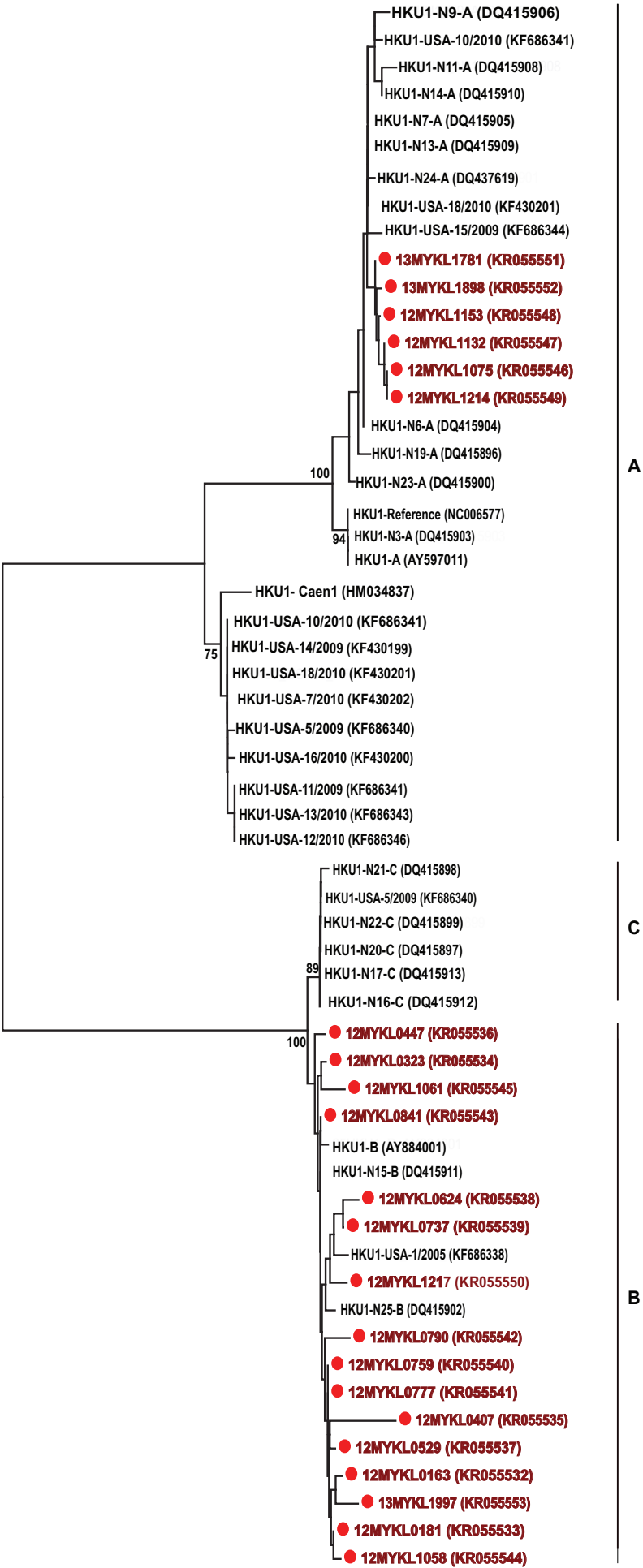

Supplement: Additional file 7: Figure S7. — Phylogenetic analysis of the HCoV-HKU1 nucleocapsid gene. Tree was reconstructed using neighbor-joining method. Bootstrap values were calculated from 1,000 trees. Bootstrap values of greater than 70 % were indicated on the branch nodes. The scale bar of individual tree was indicated in substitutions per site, using Kimura 2-parameter model in MEGA (version 5.1) to estimate pair-wise evolutionary distance. The Malaysian isolates obtained in this study were color-coded. (PDF 347 kb) [file 12985_2016_488_MOESM7_ESM.pdf]

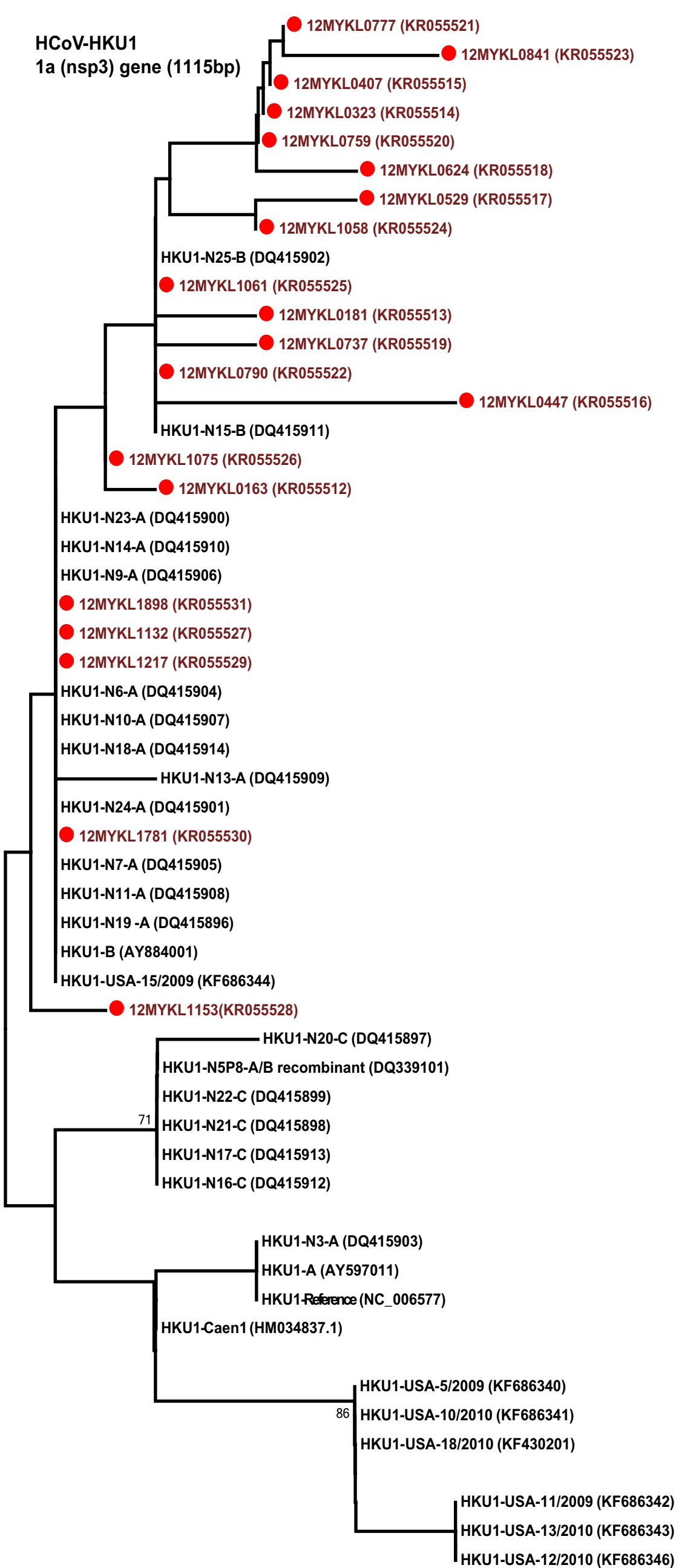

0.0005

Supplement: Additional file 8: Figure S8. — Phylogenetic analysis of the HCoV-HKU1 1a gene (nsp3). Trees was reconstructed using neighbor-joining method. Bootstrap values were calculated from 1,000 trees. Bootstrap values of greater than 70% were indicated on the branch nodes. The scale bar of individual tree was indicated in substitutions per site, using Kimura 2-parameter model in MEGA (version 5.1) to estimate pair-wise evolutionary distance. The Malaysian isolates obtained in this study were color-coded. (PDF 133 kb) [file 12985_2016_488_MOESM8_ESM.pdf]

Novel Lineage 1

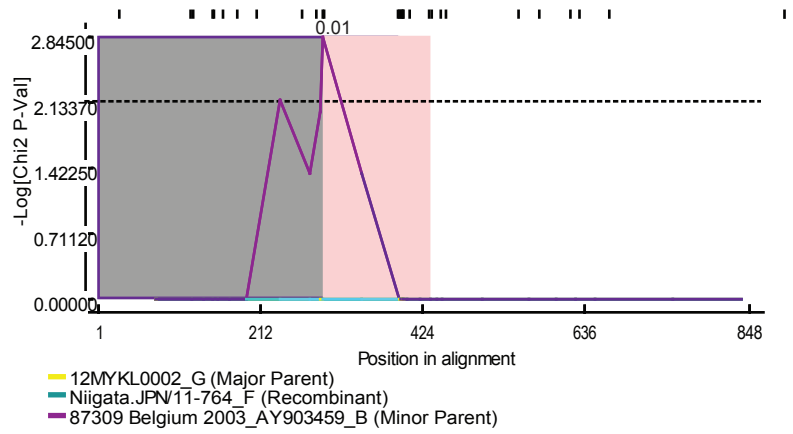

Novel Lineage 2

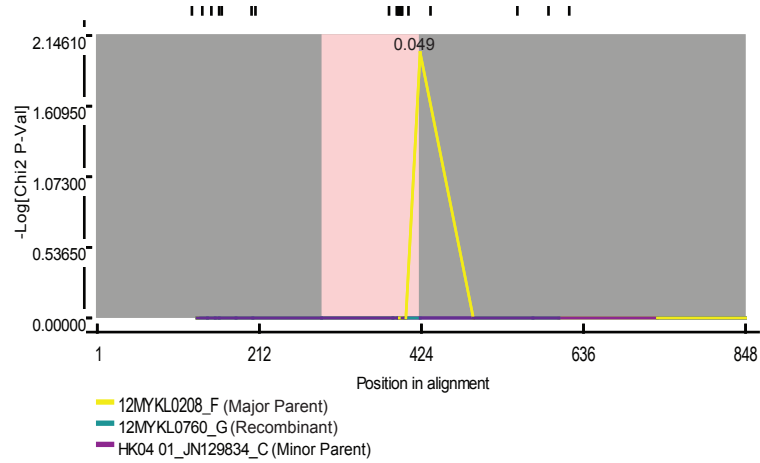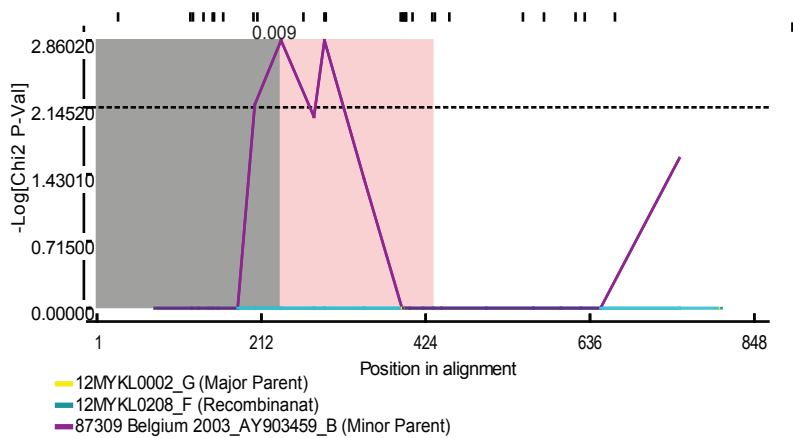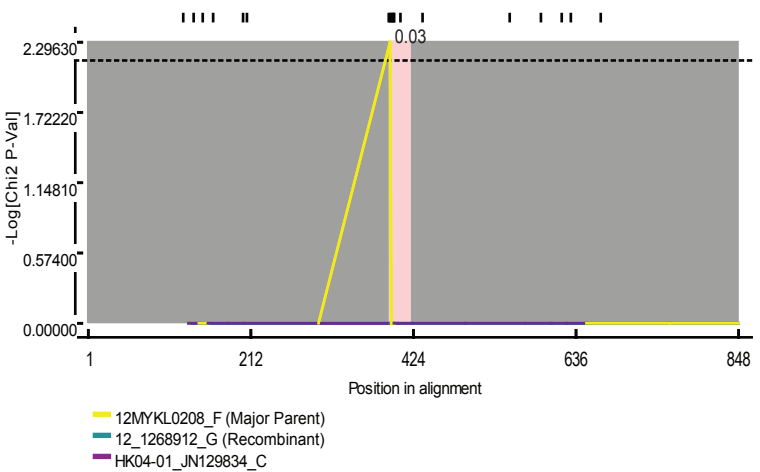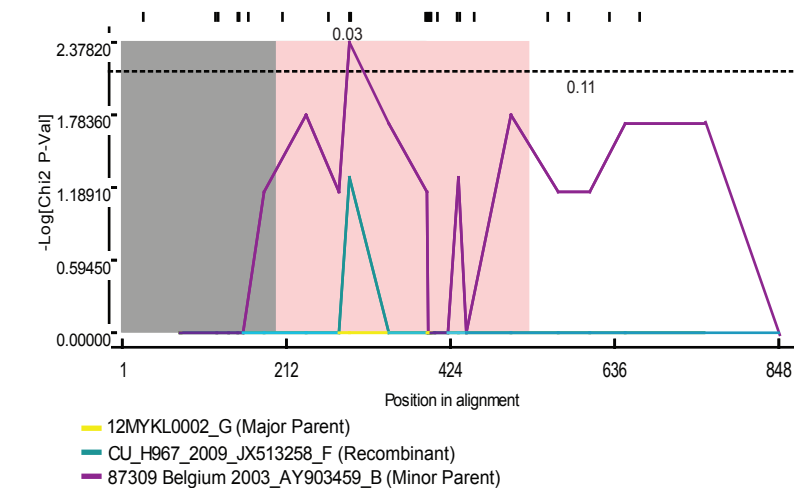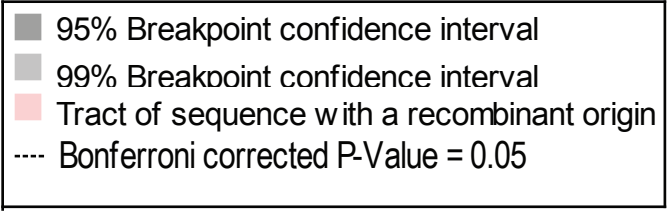

Supplement: Additional file 9: Figure S9. — Recombination analysis in HCoV-OC43 novel lineages 1 and 2. Analysis of the partial S gene was carried out using the MaxChi method in RDP. The x-axis gives the nucleotide positions of the alignment, whereas the y-axis presents the particular test statistics. Peaks in the log P of χ2 values in the MaxChi test marks potential points of recombination. Dashed lines represent p value cut-offs: uncorrected (lower line) and corrected for multiple comparisons (upper line) at the 0.05 level. (PDF 330 kb) [file 12985_2016_488_MOESM9_ESM.pdf]
